# Unmet therapeutic, educational and scientific needs in parathyroid disorders: Consensus Statement from the first European Society of Endocrinology Workshop (PARAT)

**DOI:** 10.1530/EJE-19-0316

**Published:** 2019-06-07

**Authors:** Jens Bollerslev, Camilla Schalin-Jäntti, Lars Rejnmark, Heide Siggelkow, Hans Morreau, Rajesh Thakker, Antonio Sitges-Serra, Filomena Cetani, Claudio Marcocci, the PARAT Workshop Group

**Affiliations:** 1 Section of Specialized Endocrinology, Oslo University Hospital; 2 Faculty of Medicine, University of Oslo, Oslo, Norway; 3 Division of Endocrinology, Abdominal Center, Helsinki University Hospital and University of Helsinki, Helsinki, Finland; 4 Department of Endocrinology and Internal Medicine, Aarhus University Hospital, Aarhus, Denmark; 5 Endokrinologikum Göttingen, Georg-August-University Göttingen, Göttingen, Germany; 6 Pathology, Leiden University Medical Center, Leiden, Netherlands; 7 Academic Endocrine Unit, Radcliffe Department of Medicine, University of Oxford, Churchill Hospital, Oxford, UK; 8 Endocrine Surgery Unit, Hospital del Mar, Universitat Autònoma de Barcelona, Barcelona, Spain; 9 Department of Clinical and Experimental Medicine, University of Pisa, Pisa, Italy

## Abstract

PARAT, a new European Society of Endocrinology program, aims to identify unmet scientific and educational needs of parathyroid disorders, such as primary hyperparathyroidism (PHPT), including parathyroid cancer (PC), and hypoparathyroidism (HypoPT). The discussions and consensus statements from the first PARAT workshop (September 2018) are reviewed. PHPT has a high prevalence in Western communities, yet evidence is sparse concerning the natural history and whether morbidity and long-term outcomes are related to hypercalcemia or plasma PTH concentrations or both. Cardiovascular mortality and prevalence of low energy fractures are increased, whereas quality of life is decreased, although their reversibility by treatment of PHPT has not been convincingly demonstrated. PC is a rare cause of PHPT, with increasing incidence, and international collaborative studies are required to advance knowledge of the genetic mechanisms, biomarkers for disease activity and optimal treatments. For example, ~20% of PCs demonstrate high mutational burden, and identifying targetable DNA variations, gene amplifications and gene fusions may facilitate personalized care, such as different forms of immunotherapy or targeted therapy. HypoPT, a designated orphan disease, is associated with a high risk of symptoms and complications. Most cases are secondary to neck surgery. However, there is a need to better understand the relation between disease biomarkers and intellectual function and to establish the role of PTH in target tissues, as these may facilitate the appropriate use of PTH substitution therapy. Management of parathyroid disorders is challenging, and PARAT has highlighted the need for international transdisciplinary scientific and educational studies in advancing in this field.

## Introduction

Disorders in the parathyroid glands spans a huge variety of clinical important diseases, some with a very high prevalence as in primary hyperparathyroidism (PHPT) among postmenopausal women (1, 2, 3, 4), some orphan like chronic hypoparathyroidism in adults (HypoPT) (5, 6), with increasing awareness due to new treatment algorithms (7). Parathyroid carcinomas (PC) are extremely rare, but might potentially be underdiagnosed (8, 9, 10). By far most of parathyroid diseases are sporadic as most cases of PHPT, some are part of well-known genetic conditions. As such, PHPT is linked to the multiple endocrine neoplasia (MEN) syndromes (11), and familial hypocalciuric hypercalcemia (FHH) ) is a well-described, rare autosomal dominant inherited condition most often due to a loss-of-function mutation in the calcium sensor and partner proteins (12, 13). Thus, parathyroid disorders are heterogeneous conditions with the organs (the glands) in common and with clinical awareness and interest often reflecting new developments in diagnostic procedures or treatment possibilities.

The European Society of Endocrinology (ESE) recently established a new program focusing on parathyroid disorders – the PARAT program – aiming to identify unmet scientific and educational needs in parathyroid diseases. The first workshop in Santpoort, The Netherlands in September 2018 invited European Top-experts within the three main topics, PC, PHPT and HypoPT, based on State of the Art Presentations by international experts followed by break-out sessions to identify the needs in each area and a further discussion in plenum on prioritizing the way forward in a 2- to 4-year perspective. The aim of this review is to summarize and discuss the recent achievements on these three defined topics.

## Parathyroid carcinoma

PC is one of the most rare known malignancies and accounts for less than 1% of PHPT. Reports from the United States, Australia and Finland indicate increasing incidences (8, 9, 10). Preoperatively, PC cannot be distinguished from benign causes of PHPT, as no disease-specific markers are available. PC should be suspected in patients with severe PTH-dependent hypercalcemia or its complications (14), but may be overlooked because it is so rare. PC is rather aggressive with a high recurrence rate in more than 50% of the cases and the 5-year survival rate in patients with metastatic disease is less than 50% (15, 16, 17). Patients suspected to have PC should have primary radical *en bloc* surgery, performed by an experienced surgeon (18), as this is the only treatment that can ensure cure. Diagnosis is confirmed by histopathology, which also is demanding. Therefore, some patients are correctly diagnosed only because of persistent disease after surgery or later recurrent/metastatic disease. Histopathology should always be performed by an expert parathyroid pathologist in a standardized fashion. A main challenge is to distinguish PC from an atypical parathyroid adenoma. PC is usually sporadic, but may be part of familial syndromes such as hyperparathyroidism-jaw tumour syndrome (HPT-JT), isolated familial hyperparathyroidism (FIHP) and, rarely, MEN1 or MEN2A (18, 19). *CDC73* germline mutations cause HPT-JT and, in 20–40% of the cases sporadic PC. In contrast to parathyroid adenomas, a majority of PCs are characterized by somatic *CDC73* mutations (20, 21). *CDC73* is a tumour suppressor gene that encodes parafibromin. In PC, vascular tumour invasion and loss of parafibromin expression associate with more aggressive behavior and impaired patient survival (10, 18, 22). Genetic testing should be offered to all patients with PC and when mutation positive, also first-degree relatives should be screened. For patients with inoperable disease, treatment options are limited. Treatment of hypercalcemia is crucial; however, no evidence-based disease-specific treatment exist. Given the rarity of PC, a collaborative approach is needed in order to develop novel diagnostic markers and targeted drugs, and hopefully improve outcomes.

### Preoperative diagnosis of parathyroid carcinoma

The diagnosis of PC is challenging due to the lack of reliable clinical diagnostic criteria and is in the majority of cases made postoperatively by histological examination. Local invasion of surrounding structures (trachea, inferior laryngeal nerve, strap muscles) and the occurrence of distant or lymph node metastases are the only unequivocal criteria of malignancy. The latter, however, usually develop late during follow-up. Patients who present with a palpable mass, a serum calcium level higher than 3.5 mmol/L and markedly elevated PTH levels, severe renal and/or bone disease involvement and/or laryngeal nerve palsy should be suspected of harboring PC (18). Very rarely however, PC is nonfunctioning, that is patients have normal levels of plasma calcium and PTH (23).

**Figure 1:**
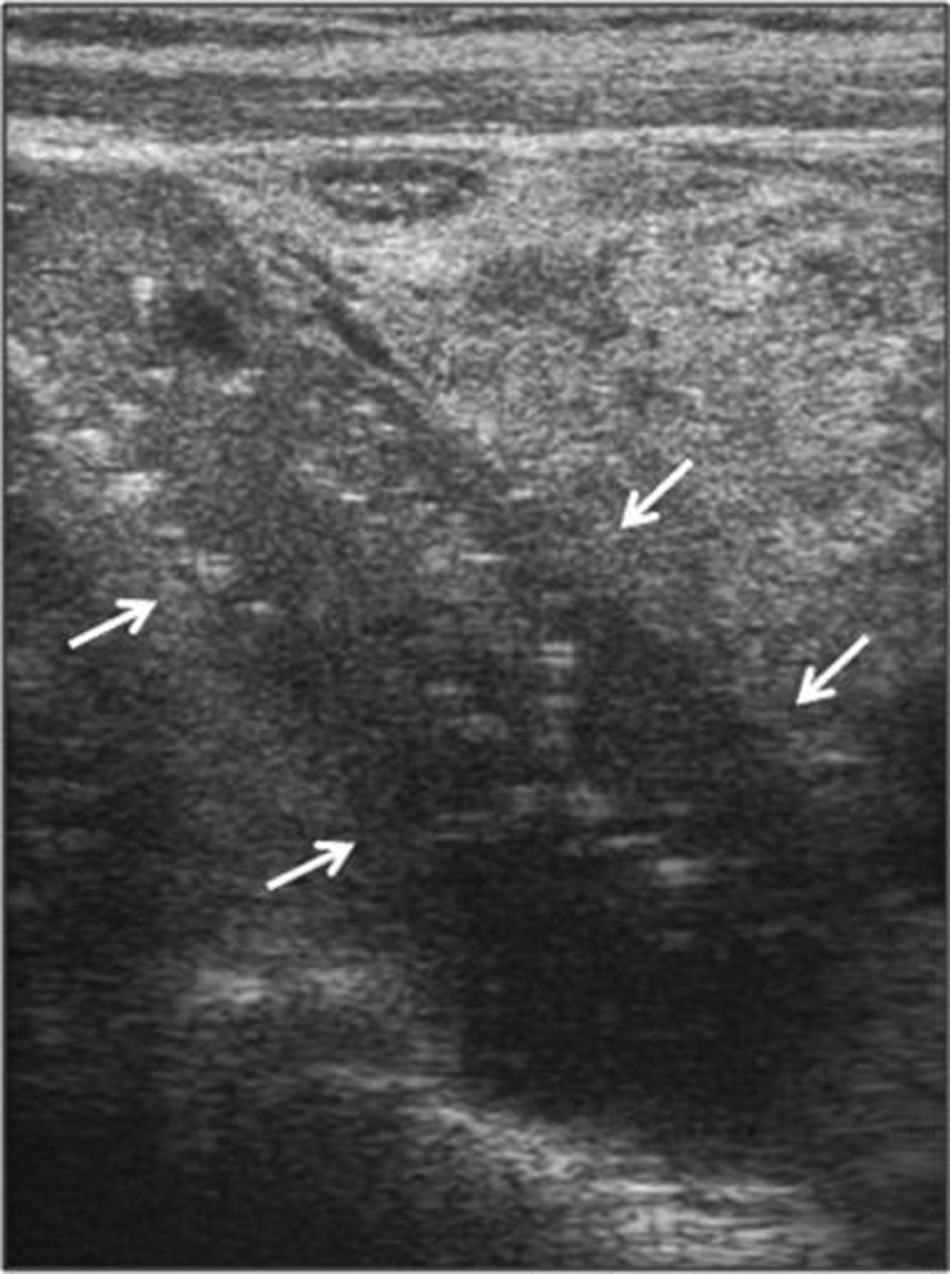
Ultrasound image of a parathyroid carcinoma. The tumor (arrows), located at the upper pole of the thyroid, shows a heterogeneous pattern, irregular shape and halo sign (longitudinal view) (from Cetani *et al*. JEI 2016, with permission (18)).

Currently, no standard imaging studies are able to distinguish PC from a benign parathyroid lesion. Nonetheless, some ultrasound (US) features may raise the suspicion for PC, namely a large (i.e. >3 cm large) lobulated hypoechoic/heterogeneous parathyroid gland, with irregular borders, thick capsule, suspicious vascularity and calcifications (24) (Fig. 1). However, these features may also be seen in benign tumors (25). Conversely, Hara *et al*. observed that the depth/width ratio of the lesion >1 suggest a PC, while <1 a benign tumor (25). Occasionally, the infiltration into surrounding tissues and cervical lymph node enlargement can be identified (or suspected). 99mTc-sestamibi (MIBI) scan is routinely used for imaging abnormal/ectopic parathyroid lesion but cannot predict malignancy. When there is a strong suspicion for PC higher resolution studies can be useful, namely contrast computed tomography (CT), including 4-dimensional computed tomography (4DCT), magnetic resonance imaging (MRI) with gadolinium or positron emission tomography (PET)/CT with 2-[fluorine-18]-fluoro-2-deoxyD-glucose (18F-FDG) (26). CT and MRI can accurately locate the lesion and its relationship with or invasion into adjacent tissues. Both are commonly used when planning *en bloc* resection of the lesion and surrounding structures. The sensitivity of a single preoperative imaging (US, CT or MIBI) has been reported to be ~80% but this increases up to 95% when the three procedures are used together (26). Thus, in a given patient with suspicion for PC combined preoperative imaging studies are recommended. The role of FDG-PET is still debated; of note, the lesions of osteitis fibrosa cystica, which can be present in patients with PC, are hypermetabolic and positive at FDG-PET and, therefore, could be misdiagnosed as bone metastasis (27). Preoperative fine-needle parathyroid biopsy is not recommended because it cannot distinguish a benign from a malignant lesion and has a risk of tumoral rupture and seeding (28).

### Pathogenesis and histopathology

#### Pathogenesis

PC can arise in the context of the *CDC73* related disorder (or HPT-JT syndrome and anecdotally in the context of MEN1 and MEN2A syndromes). Sporadic inactivation of *CDC73* and other genetic factors are driving parathyroid cancers, but there are no data why these DNA variations would occur in these relatively oligo-dividing parathyroid cells, with a relatively low chance of replication errors and less environmental influences (including lifestyle) than seen in other tumor types (29). However, a history of radiation exposure has been reported in PC (30). Proliferation of parathyroid cells due to renal failure with secondary hyperparathyroidism might occasionally drive parathyroid tumorigenesis towards malignancy (31). An alternative explanation for which there is no formal proof is that in metabolically hyperactive cells increased reactive oxygen species might lead to subsequent DNA damage targeting crucial pathways in parathyroid homeostasis. As also postulated by Tomasetti and Vogelstein for cancers in general, possibly bad luck is another valid explanation for PC tumorigenesis (29).

#### Histopathology

**Figure 2:**
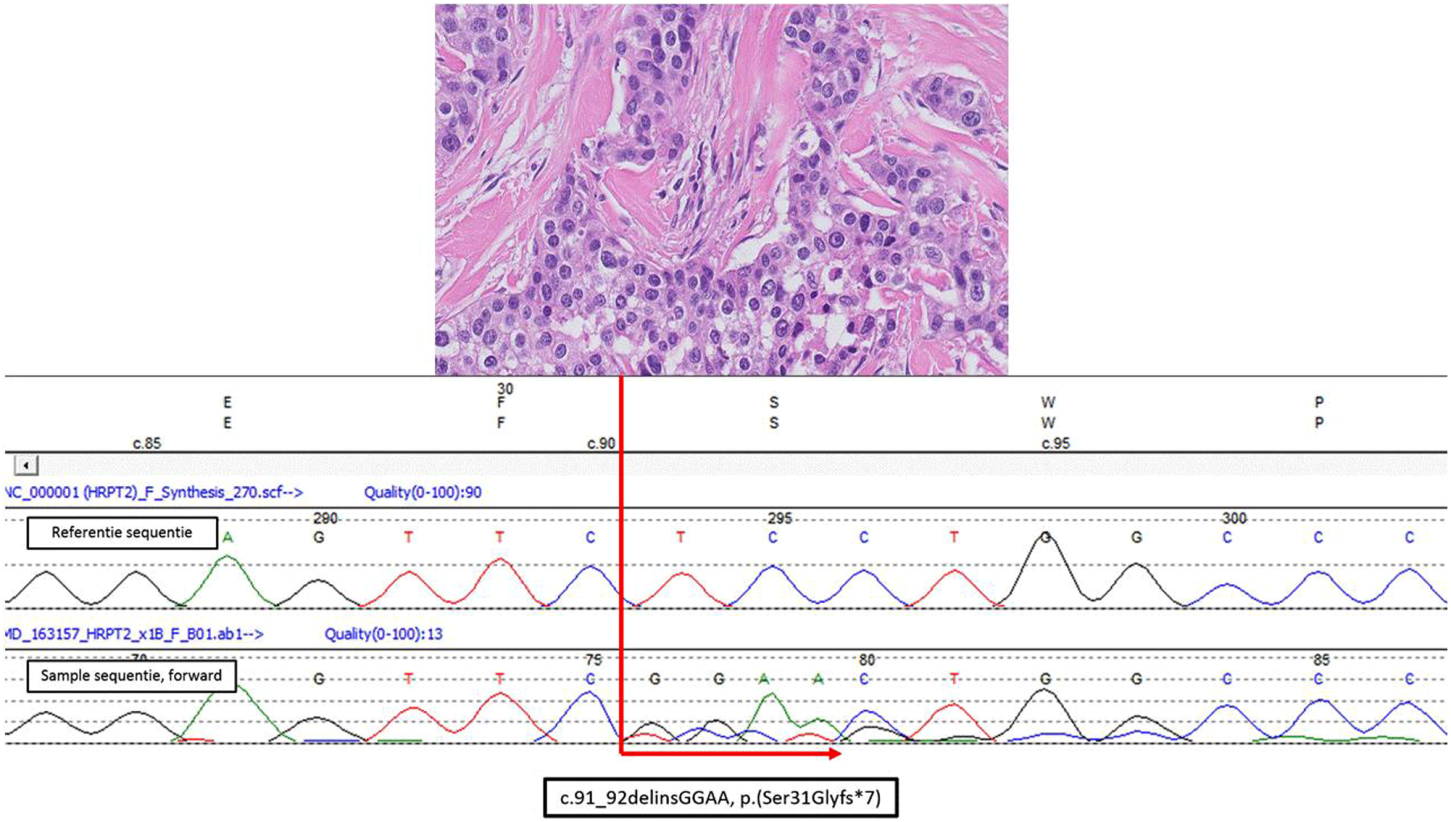
Histopathology: Parathyroid carcinoma in the context of *CDC73*-related disorder. Concerns index patient III.2 of family G described by van der Tuin *et al*. (Supplementary Fig. 1 and Supplementary Table 1 in the reference (38)). This male patient was diagnosed with PC at the age 45 years after he presented with primary hyperparathyroidism and a right-sided mass in the neck of 6 cm with lymph node metastases. The patient eventually died of this disease. Germ line *CDC73* (*HRPT2*) analysis showed a large deletion of the q-arm of chromosome 1 including the complete *CDC73* gene. In the upper part of the figure, a hematoxylin and eosin stained histological slide is presented. The tumour showed pleiomorphic parathyroid hormone positive staining cells with anisokaryosis and prominent nucleoli. Broad fibrous bands were between tumour cell islands. Occasionally mitoses were observed. The Ki-67 index was focally around 5%. Lymph angio-invasiveness outside the tumour mass was seen. Parafibromin immunohistochemistry showed vague positive and focal complete lack of nuclear staining of tumour cell in comparison with internal reference cells. There was enhanced cytoplasmic staining of tumour cells. In the lower part of the figure somatic *CDC73* (*HRPT2*) Sanger DNA-sequencing results are depicted showing a forward reference sequence above the tumour sequence. This region was not well covered in a targeted *CDC73* containing gene panel for next-generation sequencing that was also analysed. In the tumour as a second hit on the wild type allele a pathogenic *CDC73* exon 1: c.91_92delinsGGAA, p.(Ser31Glyfs*7) gene variant was identified.

The correct histopathological diagnosis of malignancy (WHO Classification of Tumours of Endocrine Organs, 2017) is primarily based on two strict criteria, that is, ingrowth of tumor cells beyond the preexistent parathyroid capsule into surrounding tissues and the presence of lymphangio-invasiveness. The presence of atypical mitotic figures and fibrous septa may be helpful as they are often associated with malignancy. An example of PC is given in Fig. 2. Other criteria like nuclear atypia and the lack of uniformity of the cells are being more circumstantial. Additional immunohistochemical staining may help to support the diagnosis and the ones that are used currently are Ki67, Galectin 3, protein gene product 9.5 (PGP9.5) and parafibromin (encoded by *CDC73*) (32). Furthermore, loss of parafibromin expression and the finding of inactivating *CDC73* DNA variants can be found in aggressively behaving parathyroid cancers (33, 34), and as such provide prognostic information.

At this moment, there are no well-established tissue biomarkers that can delineate parathyroid carcinoma (unequivocal carcinoma) from atypical adenomas (or equivocal carcinoma). For example, there is no evidence that recently described limited gene panels for PC (as outlined in the section titled 'Germline mutation testing') or larger commercial gene panels can be helpful to answer such diagnostic questions. Probably only finding *CDC73 *pathogenic variants would point to a more aggressive potential although only in cases with histological confirmation. Finding MEN1 variants would shift the odds to a more benign potential. Other biomarkers, such as microRNA profiles might be thought of.

### Germline mutation testing

Testing for germline mutation of the *CDC73* gene is strongly recommended in patients with apparently sporadic PC. The mutation detection yield ranges from 6 to 38% according to the different studies (10, 21, 33, 35, 36, 37, 38). The sample size, the selection bias and, not less important, the challenging of the histopathological diagnosis, could account for these differences. However, the latter is of utmost importance because it determines the prognosis of the patients. The majority of these mutations is widespread throughout the coding region, but most are located in exons 1, 2 and 7 (10, 21, 33, 35, 36, 37, 38). Gross deletions within the gene have been identified in a few cases of patient with PC (37, 38, 39). Therefore, in *CDC73*-negative cases for point/small mutations, the research of *CDC73* gross deletions should be considered.

Germline mutations in additional genes including *PRUNE2*, *CCD1*, *ADCK1* and genes of PI3K/AKT/mTOR pathways have been detected in patients with PC (40, 41). Therefore, gene panel testing using next-generation sequencing should be considered because it would improve the genetic analysis.

In clinical practice, the genetic testing for germline mutations is helpful in the index case and, if positive, first-degree relatives of all patients with PC in order to

1. identify the germline mutation in a given patient with PC;
2. identify asymptomatic family members carrying the mutation and thus requiring a clinical evaluation for early detection of the tumor and appropriate treatment;
3. relieve the anxiety of developing the disease family members who do not carry the mutation;
4. start the screening for HPT-JT-related tumors in the index case and healthy carriers. Indeed, patients with *CDC73*-related disorders might develop ossifying fibromas of the maxilla and/or mandible and uterine tumors and renal lesions.

### Surgery of parathyroid carcinoma

Successful surgery depends on preoperative suspicion of PC and intraoperative recognition of malignant features, such as size (often >3 cm), firmness, grayish-white color and adherence/invasion to surrounding tissues. Local excision is not acceptable, the only way of ensuring cure is complete removal of the tumor while avoiding capsule rupture and including all surrounding tissues involved (*en bloc* resection) (22). Achieving microscopically disease-free margins improves disease-free survival (42). Prognosis is influenced by surgeon performance (43). If PC is suspected, surgery should thus be performed by an expert parathyroid surgeon in a large-volume center. If the patient has not undergone primary *en bloc* resection and histopathological confirmation of PC comes as a surprise, timely further expert parathyroid surgery must be considered. Surgery is first-line treatment also for recurrent disease and, in selected cases repeated operations in combination with other systemic treatments may improve the prognosis (44).

### Systemic treatments

Most patients with inoperable PC ultimately succumb to severe hypercalcemia and kidney failure rather than tumour burden. The management of acute severe hypercalcemia includes intravenous saline infusion to restore fluid volume and increase urinary calcium excretion, and occasionally loop diuretics to enhance the calciuresis (16, 18, 45). However, such measures are of temporary success only, and other approaches (e.g. drugs such as cinacalcet) are required for longer-term success. Cinacalcet, a calcimimetic binding to the calcium-sensing receptor and thereby decreases PTH secretion, is a potent oral calcium-lowering agent. The starting dose is 30 mg twice daily, with dose adjustments every 2 weeks until calcium concentrations are acceptable (17). Nausea is a common side effect that may prevent the usage of higher doses. Cinacalcet can be combined with zoledronic acid (44), the most potent known bisphopshonate (4 mg i.v every 3–4 weeks) or, with denosumab, a monoclonal antibody that binds to RANKL (120 mg s.c monthly), especially in refractory cases and patients with impaired kidney function (46, 47). There are however, no evidence-based specific treatments. Anecdotal positive responses to adjuvant radiotherapy (10, 48, 49, 50), PTH immunotherapy (51, 52) and alkylating agents as *iv* dacarbazine (53) and oral temozolomide have been reported (44).

### Future networking, biobanking, future joint efforts for a deeper understanding of pathogenesis, to enable tailored treatments

It is of paramount importance to refer patients with suspicion of PC to tertiary care centers with skilled endocrine surgeons who are familiar with the treatment of this rare disease. It would be very desirable to set-up European prospective registries and biobanks to collect large number of patients with PC and bio-specimens (tissues, blood, serum, urine, etc). Such a project would ensure an adequate sample size to improve our understanding on not yet clear clinical preoperative, surgical and histological findings. One of the main goals would be to define preoperative biomarkers to differentiate between malignant lesions and the much more common benign counterpart, and subsequently a better understanding of the pathogenesis of PC. This would lead to better planning the initial surgical procedure as this greatly influences the prognosis of patients with PC and their management.

Extensive epidemiological data on the occurrence of PC have so far not been compiled from all countries in Europe and results on treatment outcomes have not been evaluated. Treatment options for recurrent PC are so far limited. Being a rare disease, the treatment of recurrent PC can benefit from the developments in personalized cancer care. These developments encompass the elucidation of relatively rare but targetable DNA variations, gene amplifications and gene fusions across many cancer types (54, 55). For example, DNA alterations of the PIK3CA-PTEN-mTOR-AKT pathway, gene amplifications (*HER2*, *FGFR3* etc.) or gene fusions (involving *ALK*, *ROS*, *RET*, *NTRK*) are seen in different cancer types, although with different frequencies. Indeed, recent studies reported potentially targetable *ROS1*, *TSC1*, *AKT1*, *MTOR*, *PTEN*, *PIK3CA*, *NF1*, *KDR*, *ERBB1*, *NTRK1*, *IDH1* and *FGFR3* DNA variants in PC in the presence or absence of *CDC73* mutations, with rationally matched targeted agents (41, 56, 57). Single PC cases showed clinical benefit from tyrosine kinase inhibitors (56). In PC cases with high mutational loads or burdens, the applicability of different forms of immunotherapy (for example anti PD1 immune de-blockade therapy) might be envisioned. Although the median total mutational burden in PC is relatively low (1.7 mutations per megabase (m/Mb), about 20% demonstrated high mutational burden (>20 m/Mb)) (40, 56). The latter translates into a higher neo-antigen load with a chance that abnormal tumor peptide fragments are presented on the tumor cell surface in the context of HLA and thereby leading to cytotoxic T-cell responses (58, 59). Repurposing existing drugs can be thought of as shown by the effectivity of temozolomide in an isolated PC case (44). As stated, there is a relatively high frequent inactivation of *CDC73* in PC. The parafibromin protein encoded by *CDC73* is part of the PAF1 complex involved in cell cycle regulation. It should be studied whether the latter would impose increased sensitivity to cell cycle inhibitors. Similarly cyclin D1 might be targeted as *CCND1* is found to be amplified in PC cases, reviewed recently by Costa-Guda (60). In human cancers with P16 loss (encoded by *CDKN2A*), cell cycle inhibitors are given (CDK4/6 inhibitors) (61). To bring this field further, PC-specific comprehensive integrative molecular analysis (of the mutome, transcriptome, methylome, miRNAome, etc.) is lacking. So far, isolated relatively small series of PC have been studied by extensive genomic analysis including exome analysis (40, 41, 56, 62, 63). There are no well-characterized cell lines available to facilitate biological research. For this purpose, future networking is of utmost importance to improve research and the treatment possibilities in recurrent PC.

## Chronic hypoparathyroidism in adults

Hypoparathyroidism (HypoPT) is a rare disease with an estimated prevalence of ~30 patients per 100,000 people (64). In adults, chronic HypoPT is most often secondary to neck surgery, having a surprisingly high prevalence of 10–24/100.000 inhabitants (6, 64, 65). From a therapeutic aspect, HypoPT is the last endocrine deficiency syndrome not replaced by the lacking hormone in standard care (7, 66). The indirect standard treatment or an un-physiologic substitution therapy might give challenges affecting quality of life (QoL) for the patients but also potential long-term complications (6, 67, 68, 69, 70, 71).

### The diagnosis of hypoparathyroidism

HypoPT has been clearly defined in the guidelines on 'Treatment of chronic hypoparathyroidism in adults' by ESE as ‘a disease with hypocalcaemia and inappropriately low parathyroid hormone (PTH) levels’ (7). A similar definition has been suggested by other expert groups (66, 72). Despite this general consensus, it seems that HypoPT is defined in many different ways in the scientific literature. In 2010, Mehanna *et al*. (73) published a review on papers reporting risk of postoperative hypocalcemia. Based on a systematic literature search, a total of 62 papers were identified. The majority of the papers did not specifically define hypocalcemia and the papers reporting this used ten different definitions. By applying these 10 different definitions to the same cohort of 202 thyroid surgery cases at a single institution, it was found that that the incidence of hypocalcemia varied widely (0–46%) depending on the definition used. Most recently, another systematic literature review has been published on papers reporting on risk of permanent postsurgical HypoPT between 2010 and 2011 (74). The review identified 89 articles using 20 different definitions and reported an incidence varying from 0.0–20.2%. None of the definitions used were in accordance with the generally accepted definition. Accordingly, there is a need for standardization of the definitions used for reporting hypocalcemia rates. Otherwise, patients are not diagnosed in a proper manner and thereby may not receive appropriate treatment.

### HypoPT is a complicated disease

**Figure 3:**
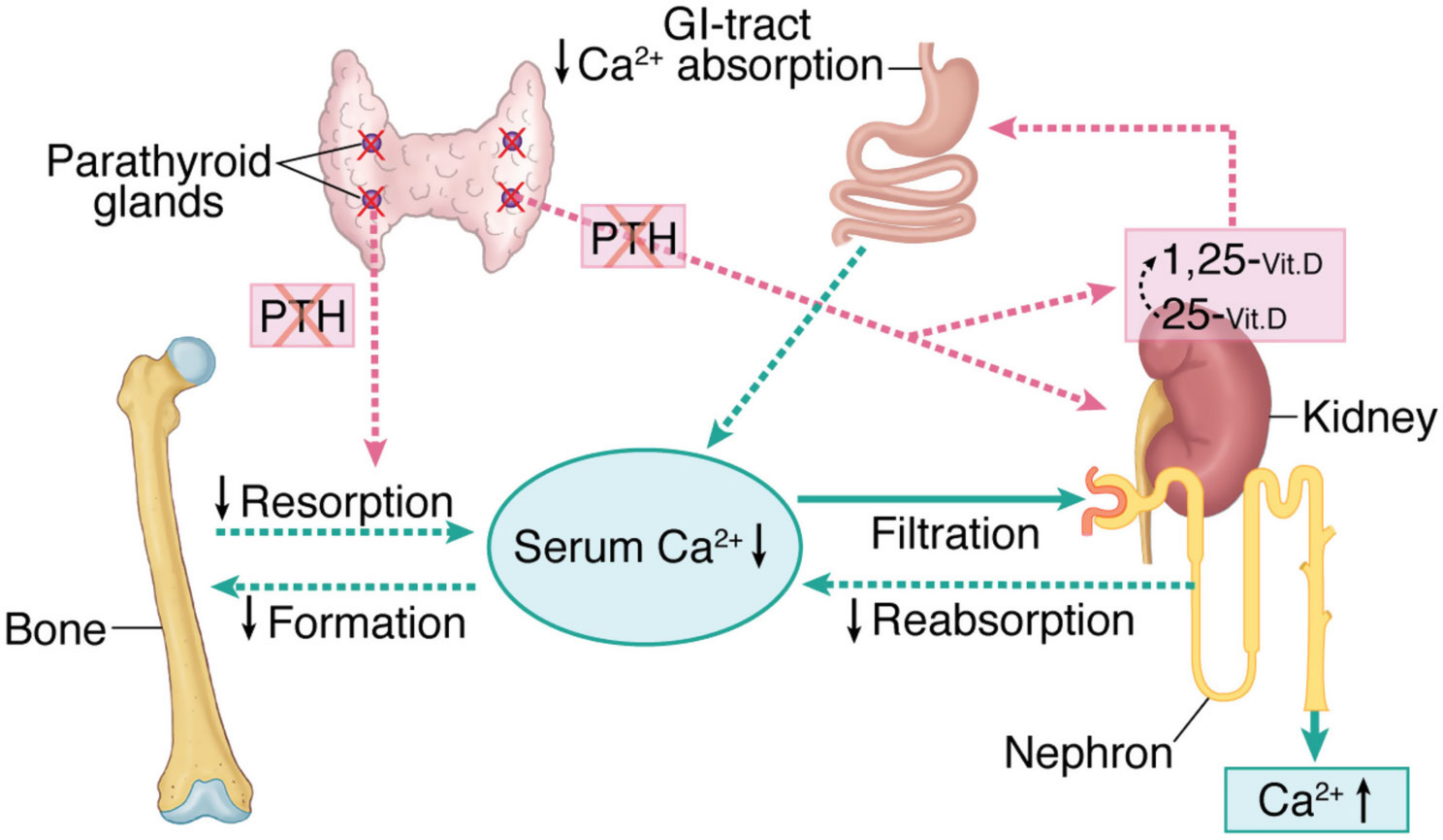
Overall calcium homeostasis in chronic hypoparathyroidism. In chronic hypoparathyroidism in adults, overall calcium metabolism is reflected by the lack of PTH. Bone turnover is markedly decreased as is the net release of calcium from the stores in bone. Levels of active vitamin D (1.25-(OH)_2_–Vit D) are low due to lack of the PTH driven 1-α-hydroxylase followed by decreased intestinal absorption of calcium. The renal tubular reabsorption of calcium is decreased. Moreover, phosphate levels are high – not illustrated in the figure.

It has become more and more clear that HypoPT is more than just a matter of supplying calcium and activated vitamin D. HypoPT is a complicated disease with deficiency of two hormones, PTH and 1,25 (OH)_2_Vit D (64) (Fig. 3). In addition to hypocalcemia, HypoPT is associated with other biochemical disturbances, such as hypercalciuria and hyperphosphatemia, as well as an impaired quality of life and an increased risk of co-morbidities, including renal stones/impairment, neuropsychiatric diseases and infections.

### Renal stones

Huge variations in the prevalence of renal stones have been reported, varying from 1.9–40%. As shown in Table 1, the presence of renal stones has been assessed by different methods, which at least in part may explain this variability. Only few studies have compared the risk of renal stones in HypoPT with the general population. A recent cross-sectional study from Italy reported renal calcifications in 30% of patients with HypoPT, which was eight-fold higher than that in an age- and gender-matched control group (odds ratio (OR) 8.2; 95% confidence interval (95% CI) 3.4–19.9) (75). On the contrary, a Danish controlled cohort study reported the risk of renal stones to be ‘only’ four-fold increased (OR 4.0; 95% CI, 1.6–9.9), compared to the general population (65). In the Italian study, renal calcifications were assessed by ultrasound examinations and most patients were asymptomatic, whereas hospital discharge codes were used in the Danish study and most patients were symptomatic. Accordingly, HypoPT seems to be associated with a four- and eight-fold increased risk of symptomatic and asymptomatic renal calcifications.

**Table 1:** Prevalence of renal calcifications in hypoparathyroidism as reported in studies from different countries.

	Country	No of patients	Type of examinations	Prevalence of renal calcifications (%)
Underbjerg 2013 (64)	Denmark	688	ICD codes	1.9
Arlt 2002 (66)	USA	25	Ultrasound	8
Rubin 2008 (152)	USA	33	?	15
Hadker 2014 (153)	USA	374	Self-reported	25
Meola 2018 (74)	Italy	82	Ultrasound	30
Lopes 2016 (154)	Brazil	40	Ultrasound	25
Mitchell 2012 (77)	USA	54	Renal imaging	31
Winer 2003 (155)	USA	27	CT scans	40

### Hypercalciuria

Idiopathic hypercalciuria occurs with a prevalence of 5–10% in the general population and is associated with an increased risk of renal stones (76). Due to the lack of PTH, renal calcium excretion is increased in HypoPT (Fig. 3) and a prevalence of hypercalciuria as high as 25–50% has been reported (75, 77, 78). Although no good data are available on associations between renal calcium excretion and the risk of renal stones in HypoPT, it seems likely to consider hypercalciuria as a causative factor for the increased risk of renal stones in HypoPT.

In general, it is recommended to aim at serum calcium levels in the low-normal range when treating HypoPT (7). The reason is the well-known association between serum and urinary calcium that is, renal calcium excretion increases with increased serum calcium levels (79). Furthermore, thiazide diuretics have been shown to decrease urinary calcium in HypoPT and may increase serum calcium levels, thereby lowering needs for calcium supplements and activated vitamin D analogues (80). In idiopathic hypercalciuria, thiazides have been shown to reduce the risk of renal stones and it seems likely that thiazides may exert a similar effect in HypoPT (81). However, the precise indication for use of thiazides in HypoPT needs further evaluations.

### Sodium restriction

Salt restriction has been associated with decreased 24-h renal calcium excretion in subjects with hypercalciuria, as a high sodium intake increases calcium excretion which also may lower serum calcium levels in HypoPT (82). Some patients with HypoPT are prone to develop sudden episodes of hypocalcemia without any obvious explanation. It should be further investigated whether an intermittent high intake of sodium may contribute to fluctuations in serum calcium levels in HypoPT.

### Biochemistry and risk of complications

In a recent study, risk of complications to HypoPT has been associated with the biochemical profile (83). Accordingly, relatively high plasma phosphate levels and/or a high calcium-phosphate product was associated with an increased mortality as well as an increased risk of renal diseases and infections. Low levels of ionized calcium were associated with an increased risk of cardiovascular diseases and episodes of hypercalcemia were associated with increased mortality and risk of infections, cardiovascular- and renal-diseases, beyond kidney calcifications. Whether improving the biochemical control can lower risk of complications of HypoPT needs to be investigated in further prospective studies.

### Are our patients well treated?

A comparison of different treatment forms in HypoPT on biochemical parameters or complications are not available to date. Treatment seems rather to depend on the availability of active vitamin D compounds in different countries. In a Danish study, alfacalcidol was exclusively used (84), in the USA calcitriol (78), in Norway both active compounds were given (6), whereas in Germany in addition to calcitriol and alfacalcidol, the long-acting dihydrotachysterol is available (67). Furthermore, the use of supplemental calcium differs geographically, being highest in the USA (78), whereas higher doses of active vitamin D are used in Europe (6, 67, 84, 85). The European guidelines and international recommendations (7, 66) propose an standardized treatment with the aim of reducing symptoms and complications by control of multiple biochemical parameters beyond serum calcium, including serum phosphate, calcium–phosphate product, magnesium and urinary calcium excretion (7). A cross-sectional study from Italy indicated that only 34.1% treated with conventional therapy (calcium and calcitriol) met the biochemical targets defined by the ESE guidelines (75). The best treatment option to reach these goals is still to be established.

### Quality of life

Optimal control of HypoPT should not be restricted simply to restoring biochemical markers. In a number of chronic diseases, quality of life (QoL) of the patients is supposed to be a hallmark of therapy (86, 87, 88) and is regarded as essential also for HypoPT patients by the ESE evidence-based guideline (7). Several instruments for the characterization of QoL in HypoPT patients were used, including the Short Form-36 (SF-36), the Hospital Anxiety and Depression Scale (HADS) and the WHO-5 Well-Being Index survey (WHO-5) (6, 68, 70, 71, 85). These different questionnaires revealed large deviations from the norms observed in the general population (6, 68, 85). However, when involving patient control groups instead of population norms studies were less encouraging (67, 69). Also treatment studies using rhPTH[1–84] using the generic SF-36 tool proved unable to detect any difference in the quality of life when compared to a control group treated with placebo (67, 69, 71). Symptoms of hypoparathyroidism are diverse and frequently hard to classify (69, 89). The knowledge and characterization of symptoms is thus essential to establish and validate tools that help to define optimal disease control (7, 90, 91, 92). Hence, the importance of hypoparathyroidism-specific instruments to assess symptoms has been regarded lately as crucial to improve our understanding of the nature and the degree of impairments to QoL (71, 90). These should also take into account patients’ compliance with treatment which may be influenced by drug cost and a multiple tablet burden. Findings from a 13-Country patient survey highlight the substantial burden of illness that negatively impacts health-related QoL (HRQoL) and health status in patients with chronic HypoPT. The degree of HRQoL reduction reflected the magnitude of symptom severity reported by patients; the greater the symptom severity level, the lower the HRQoL (93). In addition, a disease characteristic questionnaire was developed in comparison with control groups (94). With these instruments, individual differences, and their relationship to biochemical variables (if any) and treatment modalities need to be investigated in the future to identify the best treatment option for the well-being of our patients.

### Further perspectives and unmet needs in Chronic HypoPT in adults

There has been an increasing awareness on chronic HypoPT in recent years, primarily driven by epidemiologic and systematic clinical studies focusing on QoL, morbidity and co-morbidities, as indicated in the newly published treatment guidelines (7, 66). Most patients do develop chronic HypoPT following neck surgery, and there is a need for a better understanding of the pathophysiology of post-thyroidectomy parathyroid failure (95, 96). As most of the current knowledge is based on cross-sectional or short-term intervention studies, prospective studies on modern treatment modalities are warranted and needed. Such studies should be based on strict diagnostic criteria and include prospectively defined endpoint also taking comorbidities and late complications into account. PTH replacement therapy has been marketed as adjunctive treatment of adult patients with chronic HypoPT, who cannot be adequately controlled with standard therapy alone. Intuitively, substitution with the missing hormone seems an attractive approach. However, data from randomized clinical trials (RCT) have so far only documented that PTH therapy lowers serum phosphate levels and doses of calcium supplements and activated vitamin D needed to maintain normocalcemia (77, 97). Neither an improved QoL, nor lowering of urine calcium has been documented in RCTs. Currently, only treatment regimens using once-a-day injections are available. Such regimens cause a sharp rise in plasma PTH levels. However, due to a short plasma-half life, PTH is only present in the circulation for 8–12 h after an injection. Data from studies using twice-a-day injections as well as continuous infusion by pump delivery have been publish showing a plasma profile more similar to normal physiology (98). Further studies are needed to elucidate the importance of different PTH treatment regimens on its effects.

More information on transition from childhood to adult care, on pregnancy and the early puerperium is essential. QoL seems to be very low in patients with chronic HypoPT, but so far mostly based on generic questionnaires, disease specific tools should be implemented, and also studies including the burden of symptoms as perceived from the patients perspective. The prospective and randomized clinical trials with QoL as an endpoint have so far not given a clear answer on the benefit of substitution with PTH as once-a-day injections compared with standard treatment (69, 99), a further unmet need at all levels. The future development of new technology for instant calcium determination which can be used by the patients themselves would not only support the intended studies but also help to better understand the significance of value control by the individual patient on their burden of symptoms.

## Primary hyperparathyroidism

PHPT has become a common disease in developed countries, where the diagnosis often is made by chance in patients without specific symptoms, whereas the severe classical findings of PHPT most often are diagnosed in developing areas (100), potentially reflecting a real under diagnosing of the disease in these countries. With the change in clinical presentation, the question of management of asymptomatic patients (mild PHPT) has been discussed, often based on sparse evidence (101). Given PHPT as a common disease, most often in peri- and postmenopausal women (1, 2) with a prevalence of 2–5% in Scandinavian women, and most data derived from observational and case control studies, we will here concentrate on some areas of the disease spectrum that should be focused on in the immediate time to come.

### Qualitative aspects of bone tissue in PHPT

**Figure 4:**
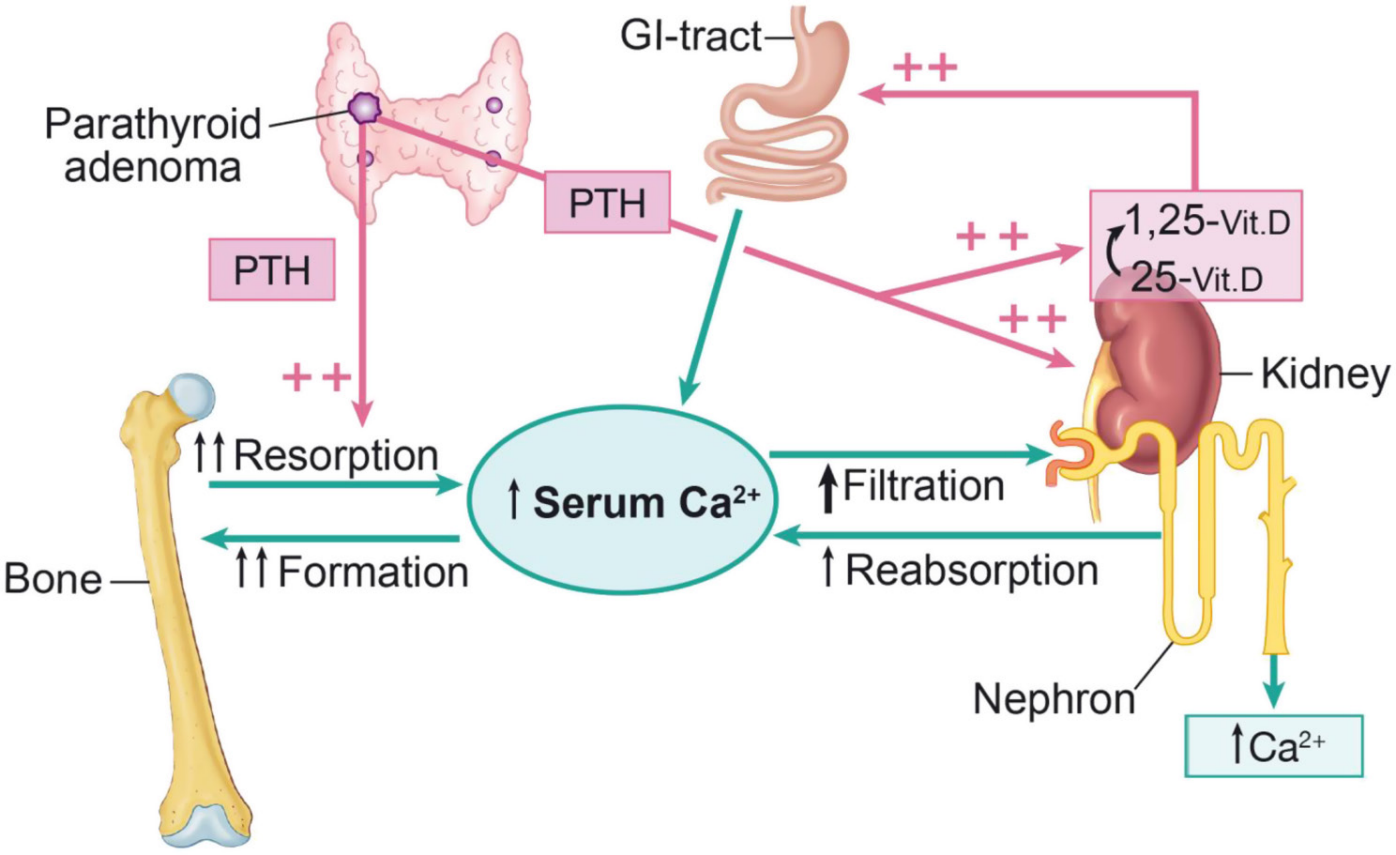
Overall calcium homeostasis in primary hyperparathyroidism. Due to a set-point error in PHPT, circulating PTH levels are inappropriately increased leading to a new steady state with increased PTH and calcium levels. Bone turnover, the renal tubular reabsorption of calcium, the activation of vitamin D followed by calcium absorption from the gut are all increased leading to the new steady state, which in principle is able to be regulated – just at a higher level.

Due to the high PTH levels (Fig. 4), bone turnover is increased in PHPT leading to an, in principle reversible bone loss based on the coupling principle and an enlargement of the remodeling space (102). Thereby, bone mass should be normalized by successful surgery and fracture frequency also be controlled. Based on large cohort studies, the fracture prevalence seemed to decrease compared to the pre-surgery situation in PHPT, but it was still increased compared the background population (103, 104). These data refer primarily to appendicular fractures, as vertebral fractures classically are underdiagnosed (101). Studies based on histomorphometry of iliac bone biopsies have indicated a preservation of trabecular bone, but also a trabecularization of the endosteal envelope leading to a thinning of the cortical shell (105, 106, 107, 108). In trabecular bone, the increased turnover is followed by an increase in the activation frequency, a shortening of the bone resorption period and of particular interest, a decrease in the resorption depth. The bone formation phase is also decreased, and the overall bone balance has been found to be even positive (reviewed in (102)). These findings are in accordance with an increased risk of peripheral fractures, but also indicating an unaltered or even reduced risk of vertebral fractures, also in agreement with findings by DXA (102). Conversely, recent studies have found a high prevalence of vertebral fractures in patients of both sexes in both younger and older patients with PHPT, and even in patients with mild PHPT (109, 110). In the recent Danish study, the overall prevalence of vertebral fractures was around 22% for all age groups, and of interest higher in men compared with women in younger patients less than 60 years. Most of the fractures were mild to moderate and only 28.7% had severe fractures by the method of Genant (111, 112). When compared to patients with postmenopausal osteoporosis, patients with PHPT seemed to fracture at a higher BMD level (112). These findings are supported by microarchitectural analyses by HRpQCT imaging, showing decreased trabecular and cortical bone microstructure in active PHPT (113, 114). Randomized controlled studies have indicated a treatment effect of surgery on bone mass by DXA in the 5-year perspective, but not for cortical bone as seen in the proximal forearm (115, 116), of importance for the risk of low energy fracture (116). Microarchitectural analyses have supported these data, showing an improvement in bone mass indices in both trabecular and cortical bone by HRpQCT, starting already 6 month after surgical cure of PHPT in both sexes, and continuing up to 2 years following surgery (117). These improvements were attributed to a decrease in bone turnover and leading to a presumably increase in bone strength in both bone compartments, as demonstrated indirectly by finate element analyses (117).

### Is the cardiovascular burden increased in mild disease and potential reversible?

Based on epidemiologic studies, PTH reflects cardiovascular (CV) risk in elderly men, even in the normal PTH range (118). PHPT is associated with a state of insulin resistance (119) and PTH is directly positively correlated to Left Ventricular Mass Index in PHPT (120). PHPT is also related to arterial hypertension, but reversibility by surgical treatment has not been demonstrated (116, 121, 122). CV-mortality is increased in PTHP and a threshold effect for calcium levels has been discussed based on case-control studies (104, 123), below which an increased CV risk could not be demonstrated. A recent meta-analysis on the effect on echocardiography measures of surgical treatment in PHPT could not demonstrate signs of reversibility neither in observational nor randomized studies (124). The findings are in accordance with biochemical markers of insulin resistance and CV-risk based on 2 and 5-year data from the randomized Scandinavian study of PHPT (121, 125). Even though a positive treatment effect of surgery on vitamin D levels (25(OH)D) was demonstrated, no effect was seen on body composition and various measures of insulin resistance 5 years following randomization (116, 125).

The increased CV-risk in PHPT is influenced by traditional CV-risk factors and these might therefore partly explain the lack of reversibility of CV-endpoints by surgical treatment in PHPT (122). At the third international workshop on management of mild (asymptomatic) PHPT, it was stated that demonstration of CV-risk reversibility by surgery could change overall recommendations (126). Even though data from long term randomized studies have now emerged, the data have not indicated a change in the recommendations for management based on CV-risk. Thus, increased CV-risk is at present not considered to be an indication for parathyroidectomy in PHPT (11).

### Will patients with mild disease improve QoL with treatment?

Patients with mild or asymptomatic PHPT have decreased QoL based on generic QoL questionnaires (2, 127, 128, 129, 130). Numerous observational studies including the most recent have indicated a positive treatment effect of surgery on QoL (131, 132, 133). Randomized controlled studies have shown ambiguous results (128, 129, 130), demonstrating no significant effect of surgical treatment on QoL in patients not fulfilling the surgical criteria for PHPT, as summarized in a recent meta-analysis (134). A disease specific questionnaire (PHPQoL) has recently been developed (135). The tool seems reliable with good internal consistency and sensitive to changes over time. It was also demonstrated that PHPQoL correlated significantly with generic, traditional questionnaires and the tool was sensitive to the degree calcium related symptoms (135). So far the experience with PHPQoL tool is limited, but a recent observational study demonstrated an improvement in PHPQoL following parathyroidectomy, independent of calcium levels (136). PHPQoL has so far not been tested in randomized, controlled studies.

Due to ambiguous results from controlled studies and few data based on disease specific questionnaires, low QoL alone has not been an indication for surgical treatment of PHPT (11).

### Normocalcemic PHPT: How can the diagnosis be secured?

Normocalcemic PHPT is defined by the finding of plasma PTH consistently (at least three determinations) elevated associated with normal total (albumin-corrected) and ionized calcium, after the exclusion of any other cause of secondary hyperparathyroidism:

1. serum 25(OH)D <75 nmol/l,
2. eGFR <60 ml/min per 1.73 m^2^,
3. 24-h urinary calcium >8.75 mmol (350 mg),
4. gastrointestinal disorders associated with calcium and/or vitamin D malabsorption, and
5. medications that could alter calcium homeostasis, including lithium, thiazide- and loop-diuretics, bisphosphonates or Denosumab (137).

These patients should be distinguished from patients with classical hypercalcemic hyperparathyroidism, who occasionally may have normal total and ionized serum calcium. We should also remind that about 10-15% of patients with classic PHPT may have albumin-corrected serum calcium in the normal range, but elevated ionized calcium (138).

Several critical issues should be considered to secure the diagnosis of normocalcemic PHPT. The first problem is the definition of normocalcemia using the range of serum calcium in the normal population (usually 2.15 – 2.55 mmol/l (8.6-10.2 mg/dl)). The individual variation in serum calcium is four times narrower that the inter individual variability, and for ionized calcium the variability is in the order of 0.02 mmol/liter (139). Thus, to be certain that the measured serum calcium is normal, we should know the range of variability of each individual subject. Therefore, facing a subject with the biochemical signature of normocalcemic PHPT, particularly when ionized calcium is in the upper normal range, we cannot exclude that the correct diagnosis could be classical PHPT, which is much more common than normocalcemic PHPT. Before making a definite diagnosis of normocalcemic PHPT, the patient must be replete for calcium and vitamin D intake for several weeks before the diagnostic sample. A second problem is the exclusion of secondary hyperparathyroidism, particularly when ionized calcium is in the lower normal range, on the basis of the cut-off values identified for serum 25(OH)D, eGFR and 24-h urinary calcium. The relationship between plasma PTH and each of these variables differs in different individuals. Therefore in the same individual, the coexistence of increased plasma PTH associated with values of serum 25(OH)D and eGFR above, and 24-h urinary calcium below the relative cut-offs, might be compatible with a form secondary hyperparathyroidism. A third problem is to exclude subtle gastrointestinal disorders that may cause calcium malabsorption. A fourth problem emerged from data from the Dallas Heart study, in which, after the exclusion of subjects with eGFR <60 ml/min and thiazide diuretics and lithium use, the prevalence of normocalcemic PHPT dropped from 3.1% to 0.6%, when many of these subjects were reinvestigate 6 years later (137). Finally, a low calcium intake could also contribute to increasing plasma PTH. In conclusion cautions should be recommended before concluding that a biochemical signature translates into a disease entity. Nonetheless, these subjects should be followed because some of them may develop the classic hypercalcemic form of PHPT (140).

### Risk factors for nephrolithiasis and value of the ‘biochemical urinary stone risk profile’

Recent studies have shown an increasing prevalence (7-35%) of silent kidney stones in patients with PHPT (112, 141). Hypercalciuria is a risk factor for nephrolithiasis and a higher mean urinary calcium excretion has been shown in patients with PHPT and kidney stones compared with those without (112). On the other hand, nephrolithiasis may occur in the absence of hypercalciuria, suggesting that factor(s) other than hypercalciuria may contribute to stone formation. In this regard, the latest guidelines for the management of asymptomatic PHPT recommend a more complete biochemical urinary stone risk profile evaluation, when 24-hour urinary calcium excretion is above 10 mmol (400 mg) (11). The rational for choosing this cut-off value is unclear and does not consider that the upper limit of normal urinary calcium excretion differs between men and women (7.5 mmol vs 6.25 mmol (300 mg vs 250 mg daily)). Tay *et al*. (142) recently reported that only 31.6% of patients with asymptomatic PHPT and occult nephrolithiasis had U-Ca >10 mmol/24 h (400 mg/24 h). Similarly, we found urinary calcium above this cut-off in 18 of 38 (47.4%) patients with asymptomatic PHPT and silent nephrolithiasis (unpublished data), confirming a poor sensitivity of this cut-off. Therefore, the question arises, whether other cut-off values could be more appropriate. Some, but not all, studies in mixed cohorts of symptomatic and asymptomatic PHPT have shown that high 24 h urinary oxalate and low citrate excretion are associated with nephrolithiasis (143). Further studies with a more complete assessment of urinary parameters that might be involved in stone risk formation in patients with asymptomatic PHPT are warranted.

### Why are International guidelines not followed as expected?

Cohort studies as well as retrospective reviews of group’s records have shown that parathyroid surgery was performed in almost half of patients with symptomatic PHPT, in 30-40% of those with asymptomatic PHPT who met at least one criterion for surgery, and in about one quarter of patients with asymptomatic PHPT who did not meet any criterion for surgery (144, 145, 146). A prospective study carried out in Italian tertiary referral centers in patients with newly diagnosed PHPT confirms that international guidelines are not obeyed over a follow-up period of one year after diagnosis. Indeed, parathyroidectomy was performed in 55.1% of patients with symptomatic PHPT and 53.7% of those with asymptomatic PHPT with at least one criterion for surgery. In both subgroups surveillance was advised when parathyroid imaging studies were negative. Parathyroidectomy was also performed in 25.7% of patients with asymptomatic PHPT and no surgical criteria, especially in those with positive parathyroid imaging studies (75%) (147). Therefore, in view of the well-established selective approach to parathyroid surgery (148), imaging studies could be considered in the therapeutic decision-making process in patients with asymptomatic PHPT, as suggested by some guidelines (149, 150).

### Further perspectives and areas of uncertainty

Although bone as target tissue has been a matter of clinical and scientific interest for decades, the biomechanical properties of bone in PHPT is still a matter of uncertainty and debate. The most recent studies have indicated increased risk of low energy fractures even in mild PHPT, but also that patients with PHPT might fracture at a higher BMD level than expected and compared with patients with postmenopausal osteoporosis (110). The continuous loss of bone mass in non-operated patients calls for consideration of a more active approach towards fracture protection, being surgery or antiresorptive therapy (115, 116), than recommended by the most recent international workshop (11). Another question arise when to start protective bone active treatment even before considering surgery both in classical or mild PHPT.

Several international guidelines and consensus reports have been published on the management of PHPT, and it is worrisome that the recommendations often not are followed. Recent long term data have emerged from randomized studies of mild PHPT without significantly demonstrating a treatment effect by surgery on CV-risk, presumable because of the importance of other CV-risk factors. Thus, at present the proposed threshold for calcium levels in PHPT given by epidemiologic studies has not been challenged. Most probably, the increased CV-risk is a combination of traditional risk factors for CV disease, and as well the increased calcium as PTH levels (151).

QoL is another area of controversy, as patients with PHPT have decreased scores by generic QoL tools and disease specific questionnaires only recently have been developed (135). So far, no benefit of parathyroidectomy on QoL from randomized studies has been demonstrated, and data from disease specific questionnaires are warranted.

## Discussions and conclusions

The presentations and discussions from the first PARAT workshop held in Santpoort, The Netherlands in September 2018 are described in the present review, focusing on parathyroid disorders from an organ perspective with emphasis on unmet needs of importance for modern patient care.

Whereas benign parathyroid adenomas and hyperplasia are common in the clinic, PC are rarely seen and do call for special attention and transdisciplinary collaboration, the parathyroid surgeon and pathologist having central roles. The understanding of the pathogenesis and natural history of PC is virtually unknown. Due to the rareness, no center can claim high experience on PC, and there is a need for central registries at different levels, for example through the European Reference Network on Rare Endocrine Conditions (ENDO-ERN). From the perspective of the PARAT project, such a registry should have priority, but it is equally important to join forces to increase experience and knowledge, and moreover to avoid parallel registries. Lessons should be learned for improving preoperative diagnosis, but at the moment no reliable biomarker for PC has been developed or validated. A multinational registry could be a way forward, also giving possibilities for second opinion at the histopathological level, and for long-term follow-up of patients.

There is an international need for stringent diagnosing of chronic HypoPT in adults, and we suggest following the ESE definition of *low calcium levels with low or inappropriate low levels of PTH* (7), where the chronic state refers to 6-month duration, but 12 months could be considered to allow recovery (96). There is a need for a better understanding of the role of *in situ* parathyroid gland preservation in the prevention of HypoPT following surgery and to gain knowledge on the potential parathyroid reserve (95, 152). The burden of HypoPT is high for the patient with impairment in QoL and cognitive properties, often with fluctuations within short time. There is a need for better understanding of the relation between biomarkers of the disease (calcium levels) and intellectual function, but also to establish the importance of the lacking hormone (PTH) in target tissues. The optimal individual target of serum calcium concentration to be reached with therapy (within the low-normal range) may be un-physiologic and cause symptoms by some, as the individual calcium level normally varies little within a narrow range. Preoperative serum calcium concentration could guide the management of patients with post-surgical HypoPt. Most patients with chronic HypoPT are females and in fertile age raising questions about optimal guidance during pregnancy and puerperium.

PHPT is a very common disease, especially in postmenopausal women (101). At present, we know little about the natural course of the disease in the modern clinic, and again it is virtually unknown whether morbidity and long-term outcome is related to the high calcium or PTH level or both (151). Cardiovascular morbidity is increased in PHPT, but potential reversibility by surgery is still a matter of debate and an issue for further research (122). Observation without intervention has been advocated for decades, but safely for how long time still remains to be answered. The natural history of fractures in PHPT is also controversial and need to be covered (112) in prospective studies. QoL is another controversial area in PHPT, where the recent developed disease specific questionnaire might be a step forward for a better understanding of the burden of the disease (135). Several international consensus statements on the management of PHPT have been published. With the accumulation of recent knowledge, a systematic and strictly evidence based clinical guideline should be considered.

## Declaration of interest

The PARAT program of activities 2018–2019 has been supported by the European Society of Endocrinology (ESE) applying for and receiving an independent educational grant from Shire PLC. Shire has not had any opportunity to influence the agenda, planned activity schedule, choice of faculty, participants, venue, delivery formats, distribution profile of outcomes, scope of objectives or any other kind of engagement with the Steering Group or ESE Focus Area leads. 

## Funding

Further funding *RVT* is funded by a Wellcome Trust Senior Investigator Award (grant number 106995/Z/15/Z); National Institute for Health Research (NIHR) Oxford Biomedical Research Centre Program and an NIHR Senior Investigator Award (grant number NF-SI-0514-10091).
